# Space use by 4 strains of laying hens to perch, wing flap, dust bathe, stand and lie down

**DOI:** 10.1371/journal.pone.0190532

**Published:** 2018-01-05

**Authors:** Elizabeth R. Riddle, Ahmed B. A. Ali, Dana L. M. Campbell, Janice M. Siegford

**Affiliations:** 1 Department of Animal Science, Michigan State University, East Lansing, Michigan, United States of America; 2 Animal Behavior and Management, Veterinary Medicine, Cairo University, Cairo, Egypt; 3 Commonwealth Scientific and Industrial Research Organisation, Armidale, New South Wales, Australia; Gaziosmanpasa University, TURKEY

## Abstract

The laying hen industry is implementing aviary systems intended to improve welfare by providing hens with more space and resources to perform species-specific behaviors. To date, limited research has examined spatial requirements of various strains of laying hens for performing key behaviors and none has been conducted within an alternative housing system. This study investigated the amount of space used by 4 strains of laying hens (Hy-Line Brown [HB], Bovans Brown [BB], DeKalb White [DW], and Hy-Line W36) to perform 5 different behaviors in the litter area of a commercial-style aviary. Hens were recorded standing [S], lying [L], perching [P], wing flapping [WF], and dust bathing [DB] on an open-litter area with an outer perch between 12:00 and 15:00 at peak lay (28 wk of age). Still images of each behavior were analyzed using ImageJ software for 16 hens per strain, and maximum hen length and width were used to calculate total area occupied per hen for each behavior. Brown hens required, on average, 89.6cm^2^ more space for S (*P*≤0.021) and 81.5cm^2^ more space for L (*P*≤0.013) than white hens. White hens used, on average, 572cm^2^ more space to perform WF than brown hens (*P*≤0.024) while brown hens used 170.3cm^2^ more space for DB than white hens (*P*≤0.022). On average, hens of all strains were wider while perching than the 15cm commonly recommended per hen (e.g., DW: 18.03; HB: 21.89cm), and brown hens required, on average, 3.38cm more space while perching than white hens (*P*≤0.01). Brown and white hens occupy different amounts of space when performing key behaviors. These differences, along with factors such as behavioral synchrony, clustering, and preferred inter-bird distances associated with these behaviors, should be considered when creating industry guidelines, crafting legislation and designing and stocking laying hen facilities to ensure hens can fulfill their behavioral needs.

## Introduction

In the past thirty years, there has been growing interest in the behavior and welfare of laying hens from the public. A growing body of evidence suggests that hens have “behavioral needs” [1] and preventing hens from engaging in strongly motivated behaviors may reduce welfare [2]. As the laying hen industry transitions from conventional cage systems to alternative forms of housing, such as aviaries, it is important to understand hen behavioral needs, including the space required to fulfill these needs, to promote hens’ ability to fulfill them.

In a production setting, housing is one of the most influential factors impacting hen welfare. Hen housing guidelines and codes of practice frequently include recommendations on the amount of space or resource allocation per hen with the intent of allowing expression of behavioral needs such as perching, wing flapping, and dust bathing. However, relatively little research has directly examined the amount of space required for laying hens to perform these key behaviors [3–5] or postures, such as standing and lying, in which hens spend much of their day [6, 7]. Yet, if hens cannot perform these behaviors, possible results are frustration, injury, or deprivation [8]. For example, hens will work to gain perch access, particularly at night [9], and if sufficient perch space is provided for each hen, hens may spend 100% of their night perching [9, 10]. Thus, provision of sufficient perch space is a key factor in determining how many hens can roost at once and whether hens will be able to roost undisturbed. Dust bathing is also well-documented as having positive benefit for hens. This “high priority behavior” [11] is a maintenance behavior that can improve feather condition and dislodge skin parasites [12]. Regarded as a comfort behavior, it has been hypothesized that situations that do not allow hens to dust bathe may cause stress [8, 12]. While less is known scientifically about the impact of restricting wing-flapping on hen welfare, there was not physical space available to accommodate this behavior in the conventional cages that were the commercial industry’s standard for housing laying hens [2]. Concern over this restriction was a driver behind the specific mention that hens must have space to stretch their wings in many pieces of legislation or standards that have been aimed at improving hen welfare.

Aviary systems are designed to promote the behaviors described above as well as others shown by hens under extensive conditions, such as foraging, exploring, and nesting. These systems also provide more space per enclosure and per hen than the conventional cages. This larger space allowance; however, needs to be considered in the context of the presence of other birds, the frequency at which behaviors are performed, and behavioral space. Specifically, consideration must be given to flock synchrony (i.e., how many hens will engage in a given behavior simultaneously [3, 13, 14]) as well as inter-bird distance (i.e., how much space a hen will place between herself and others in her group when performing a behavior) in terms of both clustering and dispersing [6, 15, 16]. However, as a starting point for calculating how much space is needed by a flock of hens to enable the hens to perform behavioral needs while accounting for synchrony and inter-bird distance, it is essential to first understand how much actual space a hen occupies when performing various behaviors [5].

There has been limited research into hens’ use of space to perform behaviors [3–5], and even less on differences among various strains of laying hen [4]. Current information on hen space use has relied on small sample sizes, focused on a limited number of behaviors, and employed varied methodologies. For example, kinematic analysis of the space used by 9 W36 White Leghorn hen for turning (180°), lying down, and wing flapping was conducted by placing hens one at a time in a small test pen [3]. Wing flapping in this study was measured when hens jumped up to or down from perches, which may result in a different pattern and degree of wing extension compared to hens wing flapping for comfort when standing. In 2016, space occupied by Lohmann Browns and Lohmann Selected Leghorns while standing or sitting was assessed using a photo box in the home pen, with one bird measured at a time and variable numbers of hens (25–298) measured at different ages (22–72 wk) and with 3 different plumage scores [4]. A final study was conducted nearly 30 years ago [5] on hybrid Ross Brown hens in cages of varying sizes and at varying stocking densities. While these measurements may be accurate for the strains and environments in which they were conducted, it is important to further examine these behaviors in a practical environment and on several commercially popular lines of hens.

In the present study, we assessed the space required to perform 5 different behaviors (wing flapping, dust bathing, perching, standing, and lying) by 4 different strains of hens (Hy-Line Brown, Bovan Brown, DeKalb White, Hy-Line W-36). Based on differences in hen size, conformation, and activity, we predicted brown hens would occupy more space for static behaviors (standing, lying, perching) and white hens would require more space to perform dynamic behaviors (wing flapping, dust bathing). By utilizing a pixel analysis program, ImageJ, we were able to measure hens while in their day-to-day aviary housing environment.

## Materials and methods

### Ethics

All research protocols were approved by the Michigan State University Institutional Animal Care and Use Committee prior to obtaining animals and starting of the project. The approved animal use form number is 01/15-025-00.

### Hens and housing

This project studied 4 genetic strains of laying hens: Hy-Line Brown [HB], DeKalb White [DW], Bovans Brown [BB], and Hy-Line W36 (n = 576 each, n = 2,304 total) selected due to their popularity within the egg industry. Before being placed in the aviary, the hens were reared at the Michigan State University Poultry Teaching and Research Center (East Lansing, MI) in an environmentally-controlled house that contained 12 pens. Each pen held 225–250 chicks, with 3 pens per strain (n = 675–750 chicks/strain). Each pen contained feeding space and nipple drinkers consistent with industry breeder management guidelines. At 3 wk of age, chicks were able to access a wood-shaving covered floor and a roosting area. For further details on rearing of the pullets, diet, and lighting see [17].

When pullets reached 17 wk of age, they were moved into a commercial-style aviary system (NATURA60, Big Dutchman, Holland, MI) in the Laying Hen Facility at the Michigan State University Poultry Teaching and Research Center (East Lansing, MI). Birds were divided among 4 rooms, each containing 4 units (1 unit per strain in each room). Each unit was populated with 144 pullets selected at random from each of the 3 rearing pens to ensure birds were mixed. Strains were placed into units within a room in a balanced fashion so that each strain occupied a unit located in a different position across each of the 4 rooms. In total, 16 aviary units were used in this study (4 strains x 4 rooms with 1 unit per strain in each room = 16 units total).

**Fig 1:**
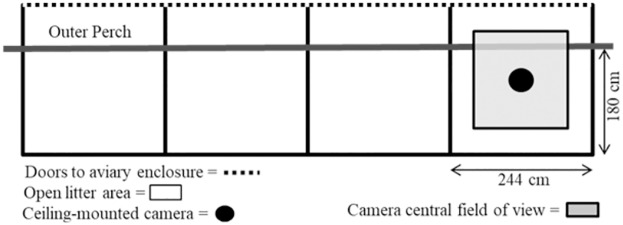
A schematic diagram of open litter areas, outer perch and ceiling-mounted cameras in aviary units. A diagram of the layout of the 4 aviary units in each room focusing on the open litter area and outer perch in front of the units. Images were captured from hens in the central field of view.

Each aviary unit consisted of 3 wire-floored tiers with an external open litter area in front of each tiered enclosure. Hens could move between the enclosure and litter area through a door on the lowest level. The litter area ran the length of each unit (244 cm wide) and was divided into an open litter area in front of the tiered enclosure (180 cm long) and a litter area underneath the tiered enclosure (163 cm long), as shown in Fig 1. A round metal perch (3.1 cm in diameter, 244 cm in length and 20 cm in height from litter floor) was positioned outside the enclosure door to help hens transition between litter and the multi-tiered enclosure, as shown in Fig 1. For this study, all images were collected from this outer perch and the open litter area. For further details on aviary design and available space per hen, see [17].

### System management and video recording

Hens did not have litter access between 17 and 25 wk of age. At 26 wk of age (when the target of *~*90% of egg production was achieved), doors on the lower tier of the aviary enclosures began opening automatically each morning at 11:30. This allowed hens daily access to litter-covered floor areas following the period of peak egg laying each day. Doors closed automatically at 01:00, approximately 5 h after lights off. Lights turned on automatically each day at 05:00 and off at 20:00. (The lights off times represent the beginning of a 30-min period of gradual overhead light dimming). For additional details on feeding, manure collection, and lighting systems see [17].

Prior to pullet placement, high-resolution digital video cameras (VF450: Clinton Electronics, Loves Park, IL) were mounted on the ceiling over the open litter area in each unit to capture hen behavior during the light period. Cameras were positioned in the middle of each open litter area. All cameras were installed at the same distance from the litter surface. Video recording was performed during light hours at 28 wk of age (peak lay period). All data were collected from 12:00 to 15:00 since this time corresponds to the time of day when hens are on litter and likely to be dust bathing [17, 18].

At the selected period of data collection, hens were acclimated to the aviary environment, litter area and the routine egg collection and counting performed routinely by farm personnel. A production level of ≥ 90%, was recorded for the whole flock at this time and hens were in visibly good health. Non-visible health conditions such as keel bone damage might interfere with hens’ desire or ability to perform behaviors of interest, including perching and standing [19], and, potentially wing flapping. Consequently, the amount of space used by hens to perform behaviors of interest might also be impacted. Therefore, at 28 wk of age, prior to data collection, 10% of hens within each unit were randomly selected and their physical health assessed following a modified version of the Welfare Quality^®^ scoring system [20]. This included assessment of body weight, feather coverage, and keel bone damage.

### Behavior choice

The behaviors selected for this study are often used in determining space allowance guidelines for housing systems, perching in particular, because they are believed to be important to hens. In 2016, [4] separated behaviors by the type of space they required: body and behavioral. In the present study, we examined behaviors within both of these categories with ‘lying’, ‘standing’, and ‘perching’ representing body space, and ‘dust bathing’ and ‘wing flapping’ representing behavioral space. The full ethogram used during this study can be found in Table 1.

**Table 1:** Ethogram, abbreviation, and explanation of how each behavior of interest was measured.

Behavior	Abbreviation	Description and Measurement
**Standing**	**S**	Standing on feet with fully extended legs, no locomotion. Head held directly above the body, in a relaxed position.
		Hens standing were measured at the longest (head/breast to tail) and widest point (widest point across the body).
**Lying**	**L**	Sternal sitting or recumbency on the floor [21]. Head held above the level of the body in relaxed position.
		Hens lying down were measured across the longest (head/breast to tail) and widest points (widest point across the body) of the body.
**Perching**	**P**	Sitting while on perch, no locomotion.
		Hens perching were measured from the sternal sitting position. The longest (head/breast to tail) and widest points (widest point across the body) were measured.
**Dust Bathing**	**DB**	The manipulation of substrate with the wings, feet, tail, and/or beak while lying in the litter with some or all feathers fluffed.
		Once dust bathing was identified, the entire dust bathing sequence was observed and a still image was captured at the time of greatest area use. If at multiple time points of dust bathing, there appeared to be similar space use, multiple images were collected and measured. The image with the largest area was used for the analysis.
**Wing Flapping, Stationary**	**WF**	The repeated extension and movement of wings while standing upright in a stationary position.
		Two images were taken, one with the hen’s wings in a full forward extension (See Fig 3A) and one with her wings in a full horizontal extension (See Fig 3B). The longest and widest point was measured on each image. The greater length or width between the two images was used for the final area.

### Data collection

In the current study, we measured the space required for hens to perform the selected behaviors while in their day-to-day aviary housing environment, which was stocked following the manufacturer’s recommended stocking density. Hens were not manipulated for the purpose of obtaining measurements, but were captured on video when spontaneously performing the behavior of interest while not in direct contact with other hens (to ensure we captured the full range of motion/area occupied). Prior to selecting an instance of a behavior for measurement, the entire sequence of the behavior was watched, as well as footage before the behavior began and after the behavior concluded. Measurements were only taken from examples of behavior where other hens did not contact the hen performing the behavior. This was done to limit the influence of physical agonistic interactions before, during or after measurements were made that might have influenced how the behavior was performed.

**Fig 2:**
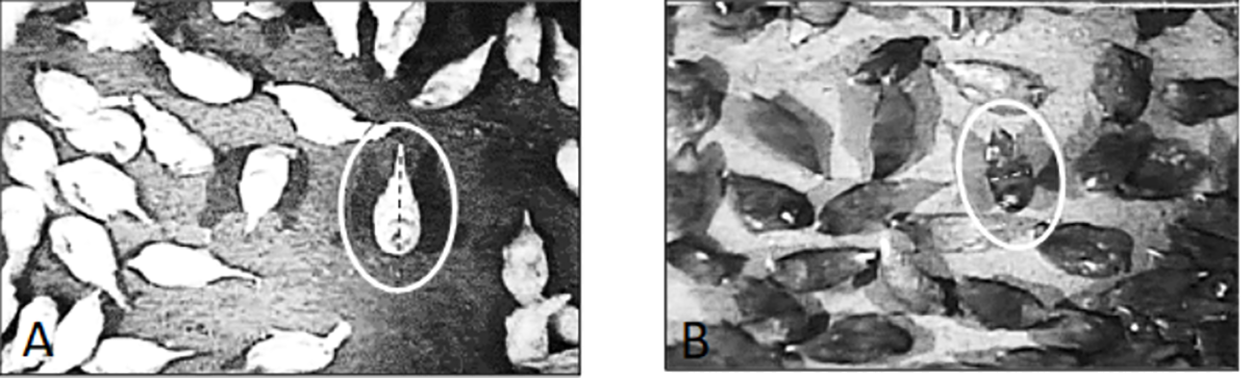
Examples of images used to measure width and length of standing hens in the open litter area. A standing Hy-Line W36 hen and a Bovans Brown hen used for measurement (circled in A and B, respectively). The lines used for measuring hen length (in A) and width (in B) are depicted.

To determine the appropriate number of observations needed for each behavior and for each strain, a statistical power analysis was conducted to estimate sample size based on preliminary data collected from 10 hens of each strain for each focal behavior (‘pwr’ package [22] in R, version 3.3.1 R Core Team, 2013). The researcher was blind to the specific strain of hen while performing measurements to minimize bias (though it was impossible to mask whether hens were brown or white). After the initial measurements were made, a power analysis was used. The power of the test was set to 80%, making the beta for this test 20% and the alpha 0.05. The standard deviation obtained from initial measurement was 115.3 cm^2^, which resulted in a sample size of n = 16 for each focal behavior per strain. To account for unit variability, four instances of each focal behavior were observed in each aviary unit; this resulted in a total of 16 images per behavior per strain. All the images collected were labeled with strain, unit, time, and date. Images of each behavior of interest within a unit were captured across the 3 h sampling interval to minimize the possibility of re-measuring the same hen. Video recordings were captured, recorded, and manipulated using GeoVision^®^ (GV-DVR/NVR, Multicam System, V15.11) with a recording rate of 30 frames per second. The scanning frequencies of the cameras were up to ≈ 15,000 Hz for the horizontal axis and ≈ 59,000 Hz for the vertical axis. These recording rates and scanning frequencies provided optimum image quality and resolution and avoided image distortion. After identifying a desired behavior in the video recordings, high quality images of the hens were captured via Snipping Tool (Microsoft Windows 10.0.15063.13 tool kit) and saved as PNG images. Images were selected for measurement when hens were in center of the litter area directly under the ceiling-mounted camera (Fig 2) to avoid any image distortion that could occur if the hen was in the outer part of the field of view. (See *Data Acquisition and Image Processing* below for more information.)

In all cases, it was important to capture the largest area occupied by the hen during the course of the behavior. For static behaviors such as standing, lying, and perching a single still image was captured. For the dynamic behaviors of dust bathing and wing flapping, the whole behavior sequence was watched before taking an image for each hen representing the largest area occupied. If it was not clear that an image did represent the largest area a hen occupied while performing a behavior, multiple images were collected from the hen performing that instance of the behavior and then measured to ensure the largest area was recorded.

### Data acquisition and image processing

**Fig 3:**
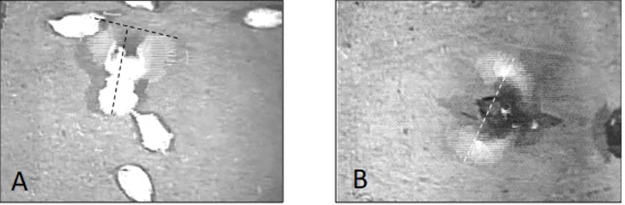
Measuring width and length of wing flapping hen in open litter area. Wing flapping showing vertical extension in DeKalb White hen (A) and showing a horizontal extension in Hy-Line Brown hen (B). The lines used to measure length (in A) and width (in B) are pictured.

Measurements from captured still images were made using ImageJ 1.50i software (Wayne Rasband, National institute of Health, USA). ImageJ has been used previously to perform accurate measurements on a variety of animals including, amphibians and caecilians and reptiles [23]. ImageJ calculated the surface area of the bird by recording the number of pixels occupied by the bird’s body after calibration of the scale to the reference object (i.e., external perch = 244 cm) in the digital images. After setting the scale between the measured pixel value of the user-defined selection and its corresponding actual length in cm, a hen’s length and width in cm were measured while performing behaviors. To measure length, a line was taken from a hen’s most distal anterior point to the most distal posterior point, and width was measured using a line across the widest part of a hen’s body, including extended wings (Fig 3).

Differences in height and angle between the reference object (i.e., the external perch) and the hen performing the behavior were corrected for to obtain accurate measurements. Objects closer to the camera appear larger and have higher pixel values when digitized compared to more distant objects. Ideally then, the test object and the pre-measured reference object should be the same distance from the camera lens. In the current study, cameras were ceiling-mounted 250 cm above the floor of the litter area, and the pre-measured reference object (i.e., the outer perch) was 40 cm height from the floor surface. However, the test objects in the current study (i.e., hens performing behaviors of interest) were at different heights from the floor relative to the outer perch, and therefore were also different distances form the camera. To account for such differences between the reference and test objects, pictures of the reference object were captured when the outer perch was placed at different distances from the floor (5 to 70 cm, in 5 cm increments). Using ImageJ, pixel values of the outer perch length at each different height was calculated, and as expected, pixel values of the outer perch increased as distance from the floor increased (i.e., as the perch was moving closer to the camera). Then, with MATLAB’s regression model, the test object’s (outer perch) position, its corresponding measured pixel value, and its known length (244 cm) were used to create a correction factor to correctly determine the actual length and width of the future test objects (i.e., hens performing behavior) at various distances from the floor of the aviary litter area. The correction factor used was: *C = B*_*0*_ *+ B*_*1*_*(H)* where C is corrected length or width (distance in cm), H is distance of the hen from the floor while performing the behavior, *B*_*0*_ (y-intercept) = 364.035, and *B*_*1*_ (slope) = 1.726.

A second procedure was used to avoid the effect of distortion caused by the location of the test object relative to the camera’s field of view. First, an initial image of the litter area was captured from each camera to visualize the camera’s field of view, and the exact central point of the field was identified (i.e., the point equidistant from all sides of this image). This central point was given a 0° angle. Images were then captured of a pre-measured box of known dimensions placed in locations on the floor of the litter area representing different angles in the camera field (0 to 60°, in 5° increments on both the X and Y axes). These images were then digitalized using ImageJ, and the pixel values of the pre-measured object at each of the different angles calculated. Pixel values of the pre-measured object were constant when measured at angles between 0 to 35°, but became distorted when at angles greater than 40°. Therefore, images of hens were only selected for measurement from areas corresponding to 0 to 35° in the field of view (i.e., almost directly under the ceiling-mounted camera as shown in Fig 1).

### Statistics

Statistical analyses were performed using the R software ‘stats’ package (version 3.3.1, R Core Team, 2013). Descriptive statistics were calculated using the “psych” package. Evaluation of data with a Shapiro-Wilk’s test (*P*>0.05) using “shapiro.test” package and a visual inspection of histograms using “hist.” package revealed that data from all measurements were normally distributed. All comparisons among strains were then performed using one-way ANOVAs using “car” package, with strain as the main effect and unit as a random effect. *P* ≤ 0.05 was considered significant. Statistically significant effects were further analyzed using Tukey’s honestly significant difference (HSD) multiple comparison procedure using the “multcomp” package [24]. Tukey’s HSD significant differences between pairwise comparisons are indicated in figures or tables by different superscript letters. Data are presented as mean ± standard error of the mean (SEM) with P values of the overall ANOVAs for each comparison.

The Intraclass Correlation Coefficient for measuring agreement (ICC) was used following [25] to measure intra-observer reliability for the same assessor for 10% of measurements for each behavior per strain using “‘ICC {cran}” package. Intra-observer reliability was calculated during the training period with the observer re-measuring the same birds twice in a random order, and a strong ICC of 0.98 (CI = 0.898) was found.

Finally, area graphs visualizing differences in space per hen (area), length and width for each behavior and between different strains were generated using MATLAB (MATLAB and Statistics Toolbox Release 2012, The MathWorks, Inc., Natick, MA).

## Results and discussion

### Hen body weights, feather cover and keel damage

Average body weights at the time of the study, when hens were 28 wk of age, were similar for hens of the 4 strains, (*P* = 0.11; DW: 1.61 ± 0.31, HB: 2.06 ± 0.29, BB: 1.98 ± 0.21, and W36: 1.58 ± 0.42 Kg). At this time, most hens also had excellent feather cover, with 95–97% of hens receiving perfect plumage scores (DW: 95.52 ± 5.63, HB: 97.78 ± 4.66, BB: 96.98 ± 3.88, and W36: 96.06 ± 4.02%), and no differences were found among the strains in degree of feather cover (*P* = 0.21). Little keel damage was observed, with 90–92% of the hens assessed having undamaged keel bones (DW: 90.56 ± 1.96, HB: 92.69 ± 2.06, BB: 91.88 ± 1.75, W36: 90.98 ± 2.06%), and no differences were found in the amount of damage seen among the 4 strains (*P* = 0.19).

### Standing

**Fig 4:**
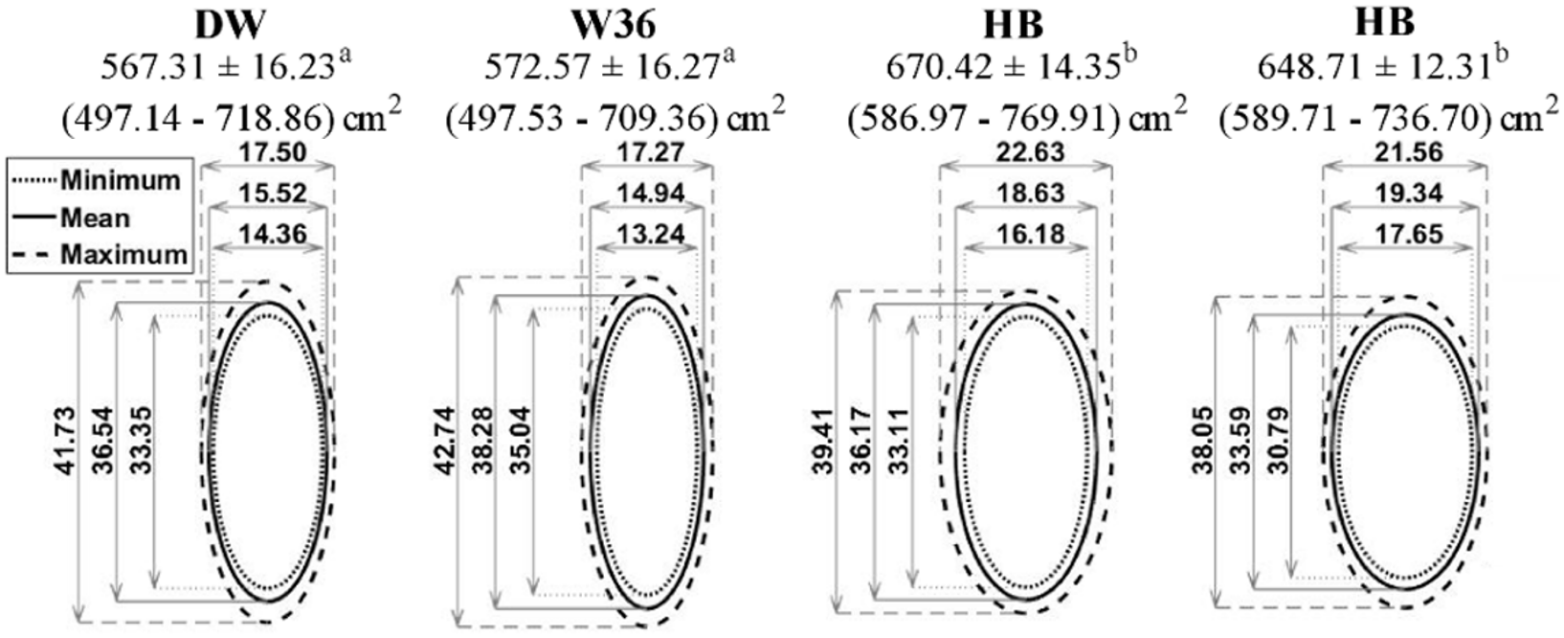
Standing space occupied by hens of 4 different strains. Standing space occupied by hens of 4 different strains presented as means ± SEM, with minimum—maximum areas in parentheses (in cm^2^). Different superscripts indicate differences (*P* < 0.05) among the standing areas occupied by different strains of hens. Ovals are used to depict minimum (dotted line), mean (solid line) and maximum (dashed line) widths and lengths (in cm) of hens of each strain while standing.

When area occupied by standing hens was compared among the 4 strains, brown hens (BB and HB) used more space than white hens (DW and W36; *P* ≤ 0.021; Fig 4). The average space occupied by standing W36 hens in the present study was 573 ± 16 cm^2^, which is similar to the 563 ± 8 cm^2^ reported by [3]. The standing spatial requirements of other strains used in this study have not been reported previously, but a similar trend of brown hens occupying more space than white hens has been described for Lohmann Brown (LB) and Lohmann Selected Leghorns (LSL) [4]. The LB hens in that study were 60 cm^2^ larger on average than the LSL hens [4], while in the present study HB and BB brown hens were between 75 cm^2^ to 100 cm^2^ larger than the DW and W36 white hens. However, though the BB and HB in the present study were similar in weight to the Ross Brown hens studied in 1989 [5], those authors reported standing space ranging from 428 cm^2^ to 592 cm^2^, which is similar to the area occupied by standing white hens in the current study. The smaller standing area reported for those Ross Brown hens [5] could be due to several factors. First, the previous study was completed almost 30 years ago, and selection pressure likely altered the size and shape of modern hens. Another possible explanation could be differences in hen age between the studies. As hens age, their feather cover and quality decreases, possibly making the space they occupy appear smaller when measured via visual assessment, as was the case in both the present and previous study. However, as the ages of hens used by [5] are unpublished, it is not possible to conclude that this is the reason for the difference between their measurements and the current ones.

### Lying

**Fig 5:**
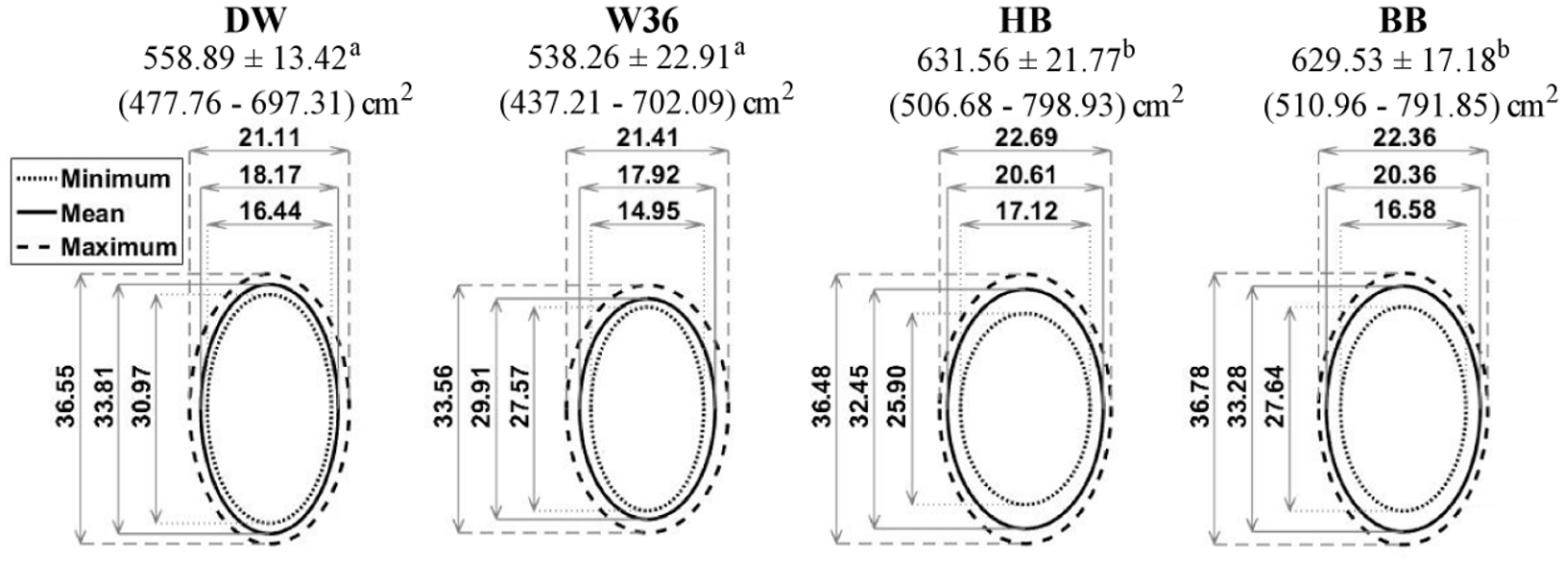
Lying space occupied by hens of 4 different strains. Lying space occupied by hens of 4 different strains presented as means ± SEM, with minimum—maximum areas in parentheses (in cm^2^). Different superscripts indicate differences (*P* < 0.05) among the lying areas occupied by different strains of hens. Ovals are used to depict minimum (dotted line), mean (solid line) and maximum (dashed line) widths and lengths (in cm) of hens of each strain while lying.

Brown hens (HB and BB) occupied more space while lying than white hens (DW and W36; *P* ≤ 0.013; Fig 5). The greatest difference in area used for lying was found between W36 and HB hens, with 93 cm^2^ more space needed on average by lying HB hens (Fig 5). Hens of all strains had relatively shorter body lengths and wider body widths when lying than standing, and this effect was more pronounced for the white hens as they had a more elliptical shape when standing.

In a previous study [3], W36 hens occupied approximately 318 cm^2^ while lying down, which is 220 cm^2^ smaller than the 538 cm^2^ reported in the current study for W36 hens. Similarly, a second study recorded LB hens using 486.70 cm^2^ and LSL using 412.84 cm^2^ when sitting [4]. These values are again smaller than those reported in the current study, but are parallel in terms of brown hens using more space to lie down than white hens. Comparing results of lying space occupied by hens in the present study to those documented in previous studies [3, 4] is somewhat difficult, as definitions of the particular type of lying or sitting/squatting behavior used for measurements were not provided [3, 4]. We defined lying as a hen resting on her breast with her head held up (i.e., in sternal recumbency). However, hens may also lay on their sides in lateral recumbency or tuck their heads under a wing when sleeping. Hens in lateral recumbency with tucked heads could present a very circular shape, which would occupy less area than the elliptical shapes we describe. Thus, differences in how lying behavior was defined among the current and previous studies may be the underlying reason for the difference in measurements, but lack of explicit ethograms prevent us from drawing a concrete conclusion to this effect. In one study, the authors referred to ‘sitting’ or ‘squatting’ rather than lying [4], which could imply sternal lying, as used in our study rather than sleeping lying posture. Because we defined lying as sternal recumbency, it is not surprising that the areas we report for lying and standing for the four strains are quite similar as the bodies and heads of the hens would be very similarly oriented with respect to the camera.

### Perching

**Fig 6:**
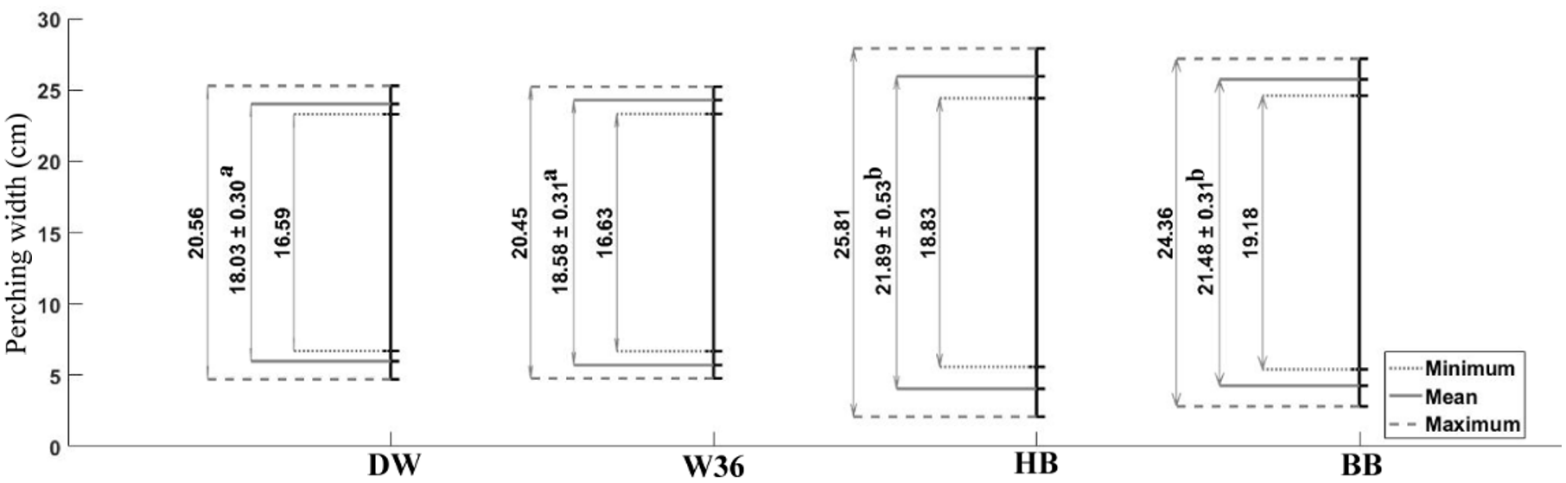
Perching width of hens of 4 different strains. Perching space occupied by hens of 4 different strains. Different superscripts indicate differences (*P* < 0.05) among the mean widths of hens of different strains of hens while perching. Lines are used to depict minimum (dotted line), mean (solid line) and maximum (dashed line) widths (in cm) of hens of each strain while perching.

As perch space guidelines are provided in linear measurements (cm or in), we assessed only the widths of hens for comparison with perching space recommendations. For example, UEP standards recommend 15 cm perch space per bird and that 50% of the flock be able to perch simultaneously on elevated perching structures [26]. In the present study, perching brown hens were wider than white hens (*P* ≤ 0.001; Fig 6), and even the narrowest individual hens measured were wider than the common 15-cm recommendation. For example, DW hens, which were the narrowest of the 4 strains assessed, had an average width of 18.03 cm, with the individual hens of this strain ranging from a minimum of 16.56 cm up to 20.56 cm in width.

Measurements in the current study were based on the visual outline of the hens, which included their feather layer. Birds in the present study were 28 wk old at peak lay period, when they had high feather quality and coverage; and hens with better feather scores have been found to occupy more floor space when standing compared to hens of worse feather scores but similar body weights [4]. Thus, some of the hen width recorded in the current study is likely due to the hens’ feather layer, and it could be argued that as this layer is compressible, it may not need to be accommodated when determining the amount of perch space physically occupied by a hen.

However, findings from previous studies [26–28] suggest that hens do prefer more than 15 cm/bird of perching space, and therefore, may not wish to compress their feather layer or pack tightly against other hens when roosting. For example, the percentage of hens perching simultaneously was found to increase from 71–78% at 15 cm/hen to 99–100% of hens perching at 22.5 cm/hen [27]. In another study [28], the authors reported the fewest hens perching at 15 cm/bird compared with treatments providing 17 to 26 cm/bird. Moreover, in a study investigating the influences of age and group size on perching behavior in a White Leghorn strain of laying hen [29], the authors calculated that 18 cm/bird was needed to allow 15- to 18-week-old laying hens to perch simultaneously.

If we consider the average width of a DW hen (18.03 cm), which were the narrowest hens in our study, and relate this to the amount of perch space available if provided at the recommended 15 cm/hen, only 83% of a flock of DW hens would actually be able to perch simultaneously. This percentage is even lower if we consider HB, the widest birds in our study, (at an average width of 21.89 cm), when only 68.5% of the hens in a HB flock could perch simultaneously if given 15 cm/hen. This difference could have a large impact on the welfare of hens. As previously stated, up to 100% of hens would prefer to perch at night [9, 10], and at least some strains of white hens prefer elevated perches at night [17], where space becomes even more limited if only 50% of the perching space provided is elevated.

### Dust bathing

Brown hens (HB and BB) used more space to dust bathe than white hens (DW and W36; *P* ≤ 0.022; Table 2); however, the average difference across all four strains was small (i.e., 188 cm^2^).

**Table 2:** Dust bathing space per hen of 4 strains.

Strain	Average Area ± SEM; cm^2^ (minimum—maximum)	Average width ± SEM; cm (minimum—maximum)	Average length ± SEM; cm (minimum—maximum)
DW	1028 ± 36.32 ^a^ (754.32–1297.94)	26.34 ± 0.86 (19.40–31.79)	39.09 ± 0.69 (34.40–44.77)
W36	1002.74 ± 28.19 ^a^ (800.81–1117.53)	24.89 ± 0.56 (21.53–31.59)	40.33 ± 0.87 (35.24–45.87)
HB	1190.99 ± 55.02 ^b^ (823.57–1694.24)	27.19 ± 1.13 (20.08–39.73)	43.75 ± 0.76 (35.29–46.73)
BB	1180.29 ± 43.04 ^b^ (950.43–1483.58)	27.98 ± 1.01 (19.40–35.05)	42.35 ± 0.93 (34.41–50.09)

Parameters are presented as means ± SEM, minimum and maximum (presented in parentheses) for area, width, and length per each strain for dust bathing space per hen of different genetic strains.

^a–b^ Means within the same column lacking a common superscript differ significantly (*P <* 0.05).

To the best of our knowledge, this is the first report of the amount of space used by hens for dust bathing. The measurements used in the present study were obtained from still images; however, dust bathing is a very dynamic behavior. Thus, the actual space used on the litter area during a bout of dust bathing would likely be greater than that reported here as hens move about while extending their wings and legs in different directions and changing the side of the body they are lying upon. Anecdotally, while collecting images during this study, some hens were in fact observed moving around the litter area as they completed a dust bathing bout. This movement around the litter area could be a reflection of other factors in a commercial aviary environment that could impact how a hen dust bathes and, thus, the true space used for dust bathing. For example, litter areas can be occupied at high rates with large numbers of hens moving around in the litter as well as between the litter areas and aviary structures [17, 18]. During times of peak occupancy or activity, this could limit the available space hens can use to dust bathe [18]. Further, the proximity of conspecifics, no matter the size of the resource, could also interrupt the behavior, making the hen move before she has completed a bout of dust bathing [13].

### Wing flapping

**Fig 7:**
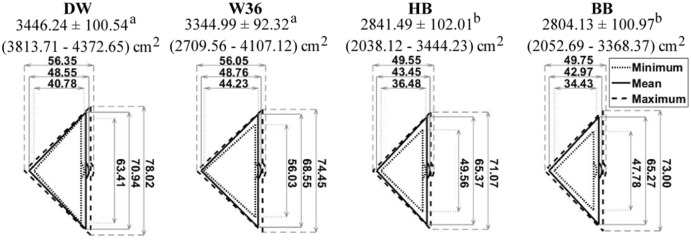
Wing flapping space of hens of 4 different strains. Space occupied by hens of 4 different strains while wing flapping. Data are presented as means ± SEM, with minimum—maximum areas in parentheses (in cm^2^). Different superscripts indicate differences (*P* < 0.05) among the wing flapping areas used by different strains of hens. Triangles are used to depict minimum (dotted line), mean (solid line) and maximum (dashed line) widths and lengths (in cm) of hens of each strain while wing flapping. See Fig 3 for a description of how lengths and widths were measured during wing flapping.

Wing flapping was the only behavior during this study where white hens (DW and W36) occupied more space than brown hens (HB and BB; *P* ≤ 0.024; Fig 7). White hens could have greater wingspan compared to brown hens, or they may extend their wings more fully when flapping. We did not measure the physical wingspan (wingtip-to-wingtip with wings manually extended by a person) of each hen that was recorded wing flapping; therefore, we are unable to speculate as to which is the cause. However, as noted previously [3], hens do not fully extend their wings when flapping; therefore it is more important to quantify the functional space required to wing flap, as we have done, rather than the physical extension of the wings.

Space needed to wing flap by hens of all strains examined here was larger than values reported previously [3, 5]. For example, W36 hens were previously reported to use an average of 1,693 cm^2^ to wing flap [3], approximately half of the 3,345 cm^2^ recorded for W36 hens in the current study while Ross Browns used 1,876 cm^2^ [5] compared to an average of 2,823 cm^2^ for the brown strains in the present study. There are several possible explanations for these differences, such as keel bone health, testing environment, and measuring methods. First, hens in current study were 28 wk of age, a full year younger than the hens used by [3]. Older hens have an increased possibility of keel bone damage [30], which could have reduced the ability or desire of these birds to fully extend their wings when flapping as keel bone fractures are painful [31].

Another possible explanation for the difference observed in space hens used during wing flapping space could be the testing environment. In the present study, hens were observed wing flapping in an open litter area of their home pen, which was a commercial-style aviary, while previous studies examined hens in a testing pen [3, 5] and in one case examined wing flapping as hens moved up to or down from a perch [3]. It is possible that hens in the test environments did not have enough space (actual or, perhaps, perceived) to extend their wings fully while flapping. Testing hens in an isolated and relatively novel environment could also possibly change the expression of wing flapping if hens were fearful or stressed to any degree. Further, wing flapping when hens are moving to or from a perch [3], may not be same as wing flapping performed as a comfort behavior. The angle and sweep of wings needed to generate upward lift or to slow a hen’s descent would be expected to present very different appearances from each other and from movements used to stretch, and may be further affected by attempts of the hen to balance while grasping a perch contrasted with a hen standing on the ground. Finally, because explanations of exactly how wing flapping was measured were not provided in previous studies [3, 5], it is possible that the forward extension of the wing during the flap was not measured or accounted for, as was the case in the current study (Fig 3). As hens move their wings forward as well as to the side while wing flapping, this forward extension must be considered because it part of the functional space needed for unrestricted wing flapping.

### Implications

In the present study, we assessed the space occupied by 4 common commercial strains of laying hens when performing behaviors in which hens spend much of their time (i.e., standing and lying) or are strongly motivated to do that promote comfort (i.e., perching, dust bathing and wing flapping). In order for poultry to perform normal behavior patterns, sufficient space is a fundamental starting point. However, while allometric principles based on the mass of an animal can help estimate body size [32], the dynamic range of motion required by a behavior must also be considered [4]. To model optimal stocking densities, at a minimum, then, the area occupied by the body of a hen when performing a behavior must be known. For poultry, the space occupied by the feather layer around the body, which could substantially increase the area occupied by the bird without increasing its mass, must also be considered as any overcrowding which compresses this layer could affect thermoregulation [33], including ability to dissipate heat, or possibly be unpleasant to the hen. Thus, we used visual measurements of the physical outline of the hens when extended to the greatest degree while performing behaviors of interest.

It is important to emphasize that the findings reported here are measurements of the space used by the hens to perform certain behaviors of interest. This does not translate necessarily into the amount of space needed by hens for optimal expression of behaviors or good welfare [4, 5]. The values reported here, and in other studies examining physical space occupancy by hens, are best regarded as a minimum baseline from which to model optimal stocking densities or enclosure sizes and configurations. Attempts to model how much space is needed by groups of hens often rest on assumptions related to how many hens would perform a particular behavior at a given time (i.e., one hen wing stretching per group of hens, with group size ranging from 5–10,000 [3]). However, while there are data related to the degree of synchrony hens show while dust bathing, feeding, laying, and perching, there are no or only limited data on synchrony of behaviors such as wing flapping, preening or foraging [34]. Thus, as estimates are made of the number of hens likely to engage in certain behaviors at any one time are made, we must be careful not to infer that birds do not want to perform certain behaviors in synchrony as it may be merely that they do not have sufficient space to show the degree of synchrony they prefer (e.g., feeding [35], nesting and litter occupancy [13], dust bathing [18]; egg laying [36]; foraging [34]). Examining degree and patterns of aggression may provide insight as to whether hens have the space needed to access resources simultaneously to the degree they wish [13].

Finally, when discussing space needed by hens to perform to behaviors, there must also be consideration of whether birds also have certain requirements for inter-bird distances when performing these behaviors that go beyond just the physical space the behavior occupies [15]. For example, hens have more inter-bird space when walking than when standing, and when pen space per hen decreases beyond a certain point hens decrease the amount of walking they perform [6]. Other behaviors, such as preening, are performed when hens are close to one another in space and do not decrease in frequency as space available per hen decreases [6, 35].

## Conclusions

In conclusion, a key finding of our study is that modern laying hens occupy more than 15 cm of perch width (Fig 6). Perching by hens at night is a highly synchronous behavior [17, 37, 38]. Sufficient perch space should be provided for all hens to roost simultaneously, and, in fact, many standards make such recommendations (e.g., [26]). Appleby [14] stated that hens accept contact with other hens while perching, but tight compression of hens on a perch can cause hens to lose balance. Therefore, in light of evidence that more hens perch simultaneously when given more than 15 cm of perch space per hen [26–28], and our assessment that even the narrowest of the hen strains we assessed occupies an average of 18 cm, perch space recommendations should be revisited. Further, as some strains of laying hens show pronounced preferences for roosting on higher perches at night [17, 37, 38], it will be important not only to consider the amount of perch space per hen, but at what height in the system this perch space is provided. As noted by Appleby [14], not only quantity, but also layout and quality of space matters in terms of animals’ ability to use it as intended.
